# Lipoprotein lipase gene polymorphisms as risk factors for stroke: a computational and meta-analysis

**DOI:** 10.22038/IJBMS.2018.29009.7001

**Published:** 2018-07

**Authors:** Majid Nejati, Mohammad Ali Atlasi, Mohammad Karimian, Hossein Nikzad, Abolfazl Azami Tameh

**Affiliations:** 1 Anatomical Sciences Research Center, Kashan University of Medical Sciences, Kashan, Iran

**Keywords:** Computational biology, Genetic polymorphism, Lipoprotein lipase, Meta-analysis, Stroke

## Abstract

**Objective(s):**

Stroke is the most common neurological disorder and genetic susceptibility has an important role in its etiology. Polymorphism in several genes such as lipoprotein lipase (*LPL*) is propounded as a risk for stroke. This meta-analysis investigated the association of rs285 and rs320 LPL polymorphism with stroke risk.

**Materials and Methods:**

We searched PubMed, Clarivate Analytics Web of Science, Google Scholar, and Science Direct databases for appropriate studies. The odds ratios (ORs) with 95% confidence intervals (CIs) were calculated to estimate the strength of this association. Also, the effects of four common polymorphisms (rs268, rs285, rs320, and rs328) on the molecular aspects of LPL were evaluated by *in silico* tools. Five studies were included in meta-analysis after screening.

**Results:**

Our data indicated that rs320 significantly decreased the risk of stroke (G vs. T: OR= 0.64, 95%CI=0.54-0.76; GG vs. TT: OR=0.47, 95%CI=0.29-0.75; TG vs. TT: OR=0.65, 95%CI=0.53-0.80; TG+GG vs. TT: OR=0.62, 95%CI=0.51-0.75; GG vs. TT+TG: OR=0.51, 95%CI=0.32-0.82). Moreover, a significant association between rs285 and diminution of stroke risk was seen (P- vs. P+: OR=0.72, 95%CI=0.58-0.91; P-P- vs. P+P+: OR=0.50, 95%CI=0.31-0.82; P+P-+P-P- vs. P+P+: OR=0.72, 95%CI=0.53-0.96; P-P- vs. P+P++P+P-: OR=0.581, 95%CI=0.369-0.916). Also, the same results were observed after stratifying, without any publication bias (*P*_Egger_>0.05). Furthermore, computational analysis revealed that rs268 and rs328 may affect the protein structure (prediction: non-neutral; score=19; expected accuracy=59%) while rs320 could affect the RNA structure (distance=0.2264, *P*-value=0.0534; *P*<0.2 is significant).

**Conclusion:**

This meta-analysis indicated that risk of stroke was decreased in rs320 and rs285 polymorphisms in the LPL gene.

## Introduction

Stroke is the main cause of permanent disability and death in adults worldwide, and is either ischemic (85%) or hemorrhagic (15%). Based on etiology ischemic stroke includes thrombotic or atherosclerotic (50%), small artery occlusion or lacunar stroke (25%), and cardioembolic (25%) (1). Hemorrhagic stroke mostly results from rupture of blood vessels secondary to trauma or high blood pressure. Hypertension, obesity, atherosclerosis, cardiovascular diseases, diabetes, hypercholesterolemia, and smoking are the major risk factors for ischemic stroke (2-4). Although lifestyle and diet are highly effective in stroke risk, the role of genetic susceptibility has been demonstrated properly. Polymorphisms in several genes such as *PRKCH* (OMIM: 605437), *PITX2* (OMIM: 601542), *ZFHX3* (OMIM: 104155), *ALOX5AP* (OMIM: 603700), *HDAC9* (OMIM: 606543), *ALDH2* (OMIM: 100650), *NLRP3* (OMIM: 606416),* MMP9* (OMIM: 120361), and *LPL* (OMIM: 609708) may be related to the risk of stroke (5-9).

Lipoprotein lipase (LPL) hydrolyzes triglycerides in chylomicrons and very low-density lipoprotein (VLDL) into free fatty acids and the glycerol molecule. Abnormal activity of this enzyme is related to hyperlipidemia and atherosclerosis, and therefore can increase the risk of ischemia. The human *LPL* gene is located on the chromosome 8 (8p22) with a 35 kb length, which comprises 10 exons (10). There are several variations in the coding and non-coding regions of this gene, some of which such as rs268, rs285, rs320, and rs328 are prevalent. The rs268 is located on exon 6 and results in an asparagine to serine substitution at codon 318 (Asn318Ser). The rs285 (*Pvu*II) and rs320 (*Hind*III) polymorphisms are located on introns 6 and 8, respectively. The rs328 polymorphism, located on exon 9, leads to termination codon at 447 (Ser447Ter). A meta-analysis revealed that there is a significant protective association between Ser447Ter and risk of stroke whereas there is no significant association between Asn318Ser and stroke risk (11). But, previous studies about the association of rs285 and rs320 polymorphisms with risk of stroke have limited sample sizes and inconclusive results. Therefore, in this study, we performed a meta-analysis with regard to the association of the two aforementioned polymorphisms with stroke risk that was followed by an *in silico* analysis.

## Materials and Methods

***Data sources***

The meta-analysis was performed on the studies that investigated the association of lipoprotein lipase gene polymorphisms with stroke risk. We searched the related articles, published prior to July 2017, using the following terms “cerebral infarction”, “cerebrovascular disorders”, “cerebrovascular accident”, “ischemic stroke”, or “hemorrhagic stroke”, in combination with “lipoprotein lipase”, “LPL”, “point mutation”, “polymorphism”, “single nucleotide polymorphism”, “SNP”, “*Pvu*II”, or “rs285”, “*Hind*III”, or “rs320” in Google Scholar, Science Direct, Clarivate Analytics Web of Science, and PubMed databases.

***Study selection and data extraction***

**Figure 1:**
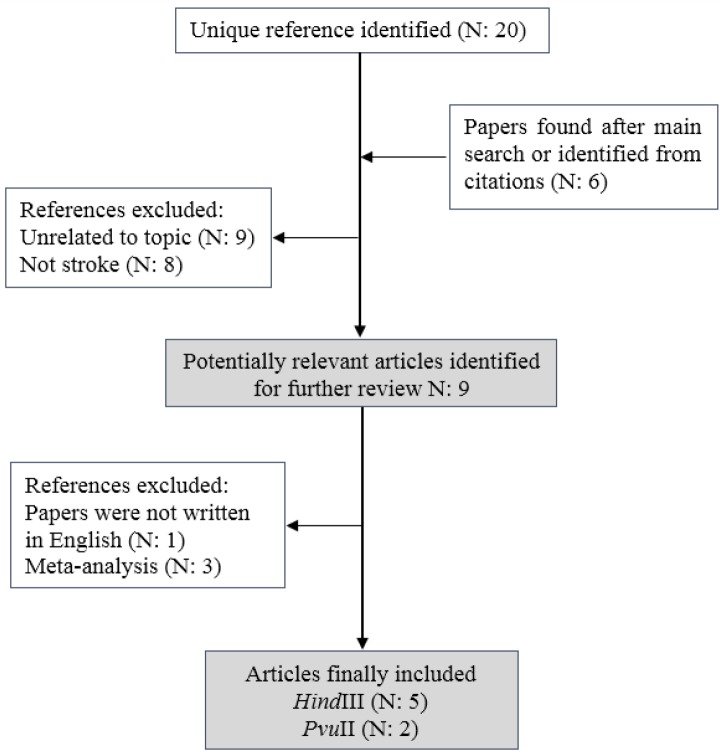
Flow diagram of the study selection process

The flowchart of the study selection procedure is presented in Figure 1. Duplicate and irrelevant papers were excluded, and abstracts of the remaining papers were then evaluated to decide whether the full-text should be gained. Related articles were selected using the following inclusion criteria: 1- Assessed the association of rs285 and rs320 SNPs of *LPL* gene and risk of stroke. 2- Case-control studies. 3- The diagnosis of stroke was confirmed by neuroimaging (CT or MRI). 4- Papers containing the data for estimation of odds ratios (ORs) and 95% confidence intervals (CIs). The following data were extracted from included studies: the names of authors, year of publication, ethnicity, genotyping method, and genotype frequencies in control and case groups.

***Statistical analysis***

At first, Hardy-Weinberg equilibrium (HWE) was calculated for control groups by the Chi-square test. The groups with a *P*-value less than 0.05 deviated from the HWE. The association of rs285 and rs320 SNPs of the *LPL* gene and stroke risk was evaluated by risk difference (RD) and odds ratios (ORs) with 95% confidence interval (CI). Meta-analysis was performed in the following models: additive, co-dominant, dominant, and recessive. The Q test and estimated *I*^2 ^score were used to calculate the heterogeneity (12), and when the *P*-value of the Q test was less than 0.1, the random-effect model was applied (13), otherwise, the fixed-effect model was applied (14). For sensitivity analysis, we excluded an individual study each time to estimate the stability of meta-analysis. Possible publication bias was evaluated by Begg’s funnel plot and Egger’s test, and a *P*-value less than 0.05 indicated the presence of publication bias (15, 16). The Open Meta-Analyst (Tufts University, Medford, MA, USA) and Comprehensive Meta-Analysis (Biostat, Inc., Englewood, NJ, USA) software packages were used for the statistical analysis. 

***In silico analysis***

The coding sequence and amino acid sequence of the *LPL* gene (Accession No. NC_000008.11) were deduced from the NCBI database. The ExPASy server (http://web.expasy.org/) was used for analysis of LPL amino acid sequence. The secondary structure of LPL was evaluated before and after Asn291Ser and Ser447Ter substitutions by Choue-Fasman, GOR, and neural network methods (http://cib.cf.ocha.ac.jp/bitool/MIX/). The three-dimensional structure of the protein was obtained from the I-TASSER server and analyzed by Accelrys DS Visualizer 4.0 (Accelrys Company; http://accelrys.com/ products/discovery-studio/visualization.php). The hydrophobic plots for the 291Asn and 291Ser phenotypes were evaluated using the Kyte and Doolittle scale (17). Also, average flexibility plots of wild and mutant types of LPL were assessed using the Bhaskaran and Ponnuswamy scale (18). The effect of Asn291Ser substitution on the function of LPL was assessed using PolyPhen-2 (http://genetics.bwh.harvard.edu/pph2/) (19) and SNAP (Screening for Non-Acceptable Polymorphisms; https://rostlab.org/services/snap/) web server (20). In addition, Net Gene2 server (http://www.cbs.dtu.dk/services/NetGene2/) (21) was applied for evaluating the effects of rs268, rs285, rs320, and rs328 polymorphisms on the splice site pattern of *LPL*. The effect of aforementioned SNPs on the RNA structure was evaluated using the RNAsnp online web server (http://rth.dk/ resources/rnasnp/) (22).

## Results

***Meta-analysis***

*Study characteristics*

According to described criteria in the methods, a total of 5 eligible articles (comprising 1160 controls and 1132 cases) for *Hind*III polymorphism and 2 article (comprising 363 controls and 362 cases) were included in the meta-analysis (10, 23-26). Flowchart of the study selection procedure is presented in Figure 1. From 9 possibly proper articles, one study was excluded because the paper was not written in English. Three other studies were excluded because they were either meta-analysis or systematic review. The key features of included studies in the meta-analysis are summarized in Table 1. Four of five articles were dedicated to the Chinese population and one of them had been studied in the Japanese population. Three studies were focused on hemorrhagic stroke and two studies were focused on ischemic stroke. The method of SNPs genotyping for all of the mentioned studies was PCR-RFLP (Table 1). 

**Table 1:** Characteristics of included studies in the meta-analysis

Country (ethnicity)	Allele frequencies				Genotype frequencies						HWE *P*^a^	Type	Genotyping method	Author and year (reference)
	Case		Control		Case			Control						
*Hind*III	T	G	T	G	TT	TG	GG	TT	TG	GG				
Chinese	645 (92.14%)	55 (07.86%)	614 (87.71%)	86 (12.29%)	301 (86.00%)	43 (12.29%)	6 (01.71%)	274 (78.28%)	66 (18.86%)	10 (02.86%)	0.019	Hemorrhagic	PCR-RFLP	Zhang *et al*. 2015 (24)
Chinese	545 (90.83%)	55 (09.17%)	515 (85.83%)	85 (14.17%)	254 (84.67%)	37 (12.33%)	9 (03.00%)	230 (76.67%)	55 (18.33%)	15 (05.00%)	<0.001	Hemorrhagic	PCR-RFLP	Xing *et al*. 2015 (10)
Chinese	218 (90.83%)	22 (09.17%)	242 (82.32%)	52 (17.68%)	99 (82.5%)	20 (16.67%)	1 (00.83%)	99 (67.34%)	44 (29.93%)	4 (02.72%)	0.735	Hemorrhagic	PCR-RFLP	Gu *et al.* 2014 (23)
Chinese	293 (79.18%)	77 (20.81%)	277 (74.46%)	95 (25.53%)	116 (62.70%)	61 (32.97%)	8 (04.32%)	103 (55.37%)	71 (38.17%)	12 (06.45%)	0.960	Ischemic	PCR-RFLP	Xu *et al.* 2008 (25)
Japanese	294 (83.05%)	60 (16.94%)	271 (76.55%)	83 (23.54%)	121 (68.36%)	52 (29.37%)	4 (02.25%)	107 (60.45%)	57 (32.20%)	13 (07.34%)	0.171	Ischemic	PCR-RFLP	Shimo-Nakanishi *et al.* 2001 (26)
*Pvu*II	P^+^	P^-^	P^+^	P^-^	P^+^P^+^	P^+^P^-^	P^-^P^-^	P^+^P^+^	P^-^P^+^	P^-^P^-^				
Chinese	245 (66.21%)	125 (33.78%)	213 (57.25%)	159 (42.74%)	85 (45.94%)	75 (40.54%)	25 (13.51%)	65 (34.94%)	83 (44.62%)	38 (20.43%)	0.228	Ischemic	PCR-RFLP	Xu *et al.* 2008 (25)
Japanese	270 (76.27%)	84 (23.72%)	253 (71.46%)	101 (28.53%)	103 (58.19%)	64 (36.15%)	10 (05.64%)	94 (53.10%)	65 (36.72%)	18 (10.16%)	0.186	Ischemic	PCR-RFLP	Shimo-Nakanishi *et al.* 2001 (26)

**Table 2:** Association results in the meta-analysis for rs320 polymorphism

Variables	*n*	G *vs*. T		GG *vs*. TT		TG *vs*. TT		TG+GG* vs*. TT		GG *vs*. TT+TG	
		OR (95% CI)	*P*	OR (95% CI)	*P*	OR (95% CI)	*P*	OR (95% CI)	*P*	OR (95% CI)	*P*
Total	5	0.64 (0.54-0.76)	< 0.001	0.47 (0.29-0.75)	0.001	0.65 (0.53-0.80)	< 0.001	0.62 (0.51-0.75)	< 0.001	0.51 (0.32-0.82)	0.005
Subtype											
Hemorrhagic	3	0.58 (0.46-0.73)	< 0.001	0.51 (0.27-0.94)	0.031	0.57 (0.43-0.74)	< 0.001	0.56 (0.43-0.72)	< 0.001	0.55 (0.30-1.03)	0.061
Ischemic	2	0.72 (0.56-0.92)	0.010	0.42 (0.21-0.87)	0.019	0.78 (0.57-1.07)	0.128	0.72 (0.54-0.98)	0.035	0.46 (0.23-0.94)	0.033
HWE											
Yes	3	0.66 (0.53-0.83)	< 0.001	0.40 (0.20-0.79)	0.008	0.69 (0.53-0.91)	0.009	0.65 (0.50-0.85)	0.001	0.44 (0.23-0.87)	0.017
No	2	0.61 (0.47-0.79)	< 0.001	0.54 (0.28-1.05)	0.068	0.60 (0.44-0.82)	0.001	0.59 (0.44-0.79)	< 0.001	0.59 (0.31-1.13)	0.112
Country											
China	4	0.63 (0.52-0.76)	< 0.001	0.53 (0.32-0.89)	0.016	0.62 (0.49-0.78)	< 0.001	0.60 (0.48-0.75)	< 0.001	0.58 (0.35-0.97)	0.039
Sample size											
<500	3	0.66 (0.53-0.83)	< 0.001	0.40 (0.20-0.79)	0.008	0.693 (0.53-0.914)	0.009	0.65 (0.50-0.85)	0.001	0.44 (0.23-0.87)	0.017
>500	2	0.61 (0.47-0.79)	< 0.001	0.54 (0.28-1.05)	0.068	0.60 (0.44-0.82)	0.001	0.59 (0.44-0.79)	< 0.001	0.59 (0.31-1.13)	0.112

OR: Odds Ratio; CI: Confidence Interval; HWE: Hardy-Weinberg equilibrium

Significant differences between the case and control groups are bolded

**Table 3:** Summary risk difference and adjusted P-value for multiple testing using the BH-FDR method

Variables	*n*	G *vs*. T			GG *vs*. TT			TG *vs*. TT			TG+GG* vs*. TT			GG *vs*. TT+TG		
		RD (95%CI)	*P*	*P*_BH-FDR_	RD (95%CI)	*P*	*P*_BH-FDR_	RD (95%CI)	*P*	*P*_BH-FDR_	RD (95%CI)	*P*	*P*_BH-FDR_	RD (95%CI)	*P*	*P*_BH-FDR_
Total	5	-0.05 (-0.07 to -0.03)	<0.001	<0.001	-0.03 (-0.05 to -0.01)	0.001	0.001	-0.07 (-0.11 to -0.04)	<0.001	<0.001	-0.09 (-0.12 to -0.05)	<0.001	<0.001	-0.02 (-0.04 to -0.01)	0.004	0.005
Subtype																
Hemorrhagic	3	-0.05 (-0.08 to -0.03)	<0.001	<0.001	-0.02 (-0.04 to 0.00)	0.030	0.039	-0.08 (-0.12 to -0.04)	<0.001	<0.001	-0.09 (-0.13 to -0.05)	<0.001	<0.001	-0.02 ( -0.03 to 0.00)	0.061	0.057
Ischemic	2	-0.06 (-0.10 to -0.01)	0.010	0.044	-0.06 (-0.106 to -0.01)	0.016	0.044	-0.06 (-0.13 to 0.02)	0.127	0.128	-0.08 (-0.15 to -0.01)	0.034	0.044	-0.04 -0.07 to -0.00)	0.028	0.044
HWE																
Yes	3	-0.06 (-0.11 to -0.02)	0.004	<0.001	-0.05 (-0.09 to -0.01)	0.006	0.011	-0.08 (-0.14 to -0.02)	0.009	0.011	-0.10 (-0.16 to -0.04)	0.001	0.003	-0.03 (-0.06 to -0.01)	0.014	0.017
No	2	-0.05 (-0.07 to -0.02)	<0.001	<0.001	-0.02 (-0.04 to 0.00)	0.066	0.085	-0.07 (-0.12 to -0.03)	0.001	0.002	-0.07 (-0.12 to -0.04)	<0.001	<0.001	-0.0154 -0.03 to 0.00)	0.107	0.112
Country																
China	4	-0.05 (-0.07 to -0.03)	<0.001	<0.001	-0.02 (-0.05 to 0.00)	0.015	0.02	-0.08 (-0.11 to -0.04)	<0.001	<0.001	-0.09 (-0.12 to -0.05)	<0.001	<0.001	-0.02 (-0.03to 0.00)	0.036	0.039
Sample size																
<500	3	-0.06 (-0.11 to -0.02)	0.004	<0.001	-0.05 (-0.09 to -0.01)	0.006	0.011	-0.08 (-0.14 to -0.02)	0.009	0.011	-0.10 (-0.16 to -0.04)	0.001	0.003	-0.03 (-0.06 to -0.01)	0.014	0.017
>500	2	-0.05 (-0.07 to -0.02)	<0.001	<0.001	-0.02 (-0.04 to 0.00)	0.066	0.085	-0.07 (-0.12 to -0.03)	0.001	0.002	-0.07 (-0.12 to -0.04)	<0.001	<0.001	-0.0154 -0.03 to 0.00)	0.107	0.112

RD: Risk difference; CI: Confidence Interval; HWE: Hardy-Weinberg equilibrium; FDR: *P*-value from Benjamini-Hochberg method control for false discovery rate

**Table 4:** Results of heterogeneity and publication bias in the meta-analysis for rs320 polymorphism

Variables	G *vs*. T			GG *vs*. TT			TG *vs*. TT			TG+GG* vs*. TT			GG *vs*. TT+TG		
	*Ph*	*I* ^2^	*P*e	*P*h	*I* ^2^	*P*e	*Ph*	*I* ^2^	*P*e	*P*h	*I* ^2^	*P*e	*P*h	*I* ^2^	*Pe*
Total	0.636	0%	0.094	0.806	0%	0.197	0.554	0%	0.251	0.643	0%	0.227	0.805	0%	0.250
Subtype															
Hemorrhagic	0.684	0%	0.030	0.802	0%	0.158	0.717	0%	0.216	0.662	0%	0.105	0.847	0%	0.167
Ischemic	0.587	0%	-	0.303	6%	-	0.862	0%	-	0.890	0%	-	0.278	15%	-
HWE															
Yes	0.315	13%	0.114	0.527	0%	0.551	0.283	21%	0.176	0.321	12%	0.037	0.514	0%	0.598
No	0.987	0%	-	0.994	0%	-	0.932	0%	-	0.962	0%	-	0.989	0%	-
Country															
China	0.477	0%	0.182	0.918	0%	0.055	0.580	0%	0.255	0.553	0%	0.261	0.938	0%	0.075
Sample size															
<500	0.315	13%	0.114	0.527	0%	0.551	0.283	21%	0.176	0.321	12%	0.037	0.514	0%	0.598
>500	0.987	0%	-	0.994	0%	-	0.932	0%	-	0.962	0%	-	0.989	0%	-

Ph, Pheterogeneity (*P*<0.1 was considered as a significant difference). Pe, Pegger (*P*<0.05 was considered as a significant difference). HWE: Hardy-Weinberg equilibrium

**Figure 2:**
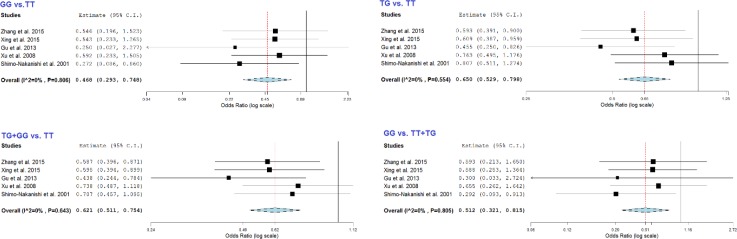
Forest plot for the association of rs320 with stroke risk

**Figure 3:**
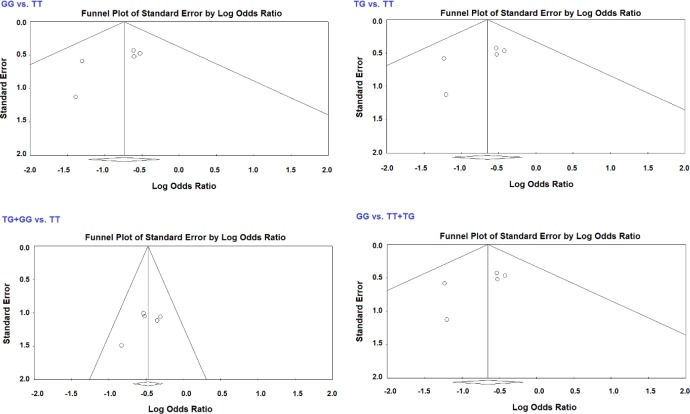
Funnel plot for the association of rs320 with stroke risk in total analysis

**Figure 4:**
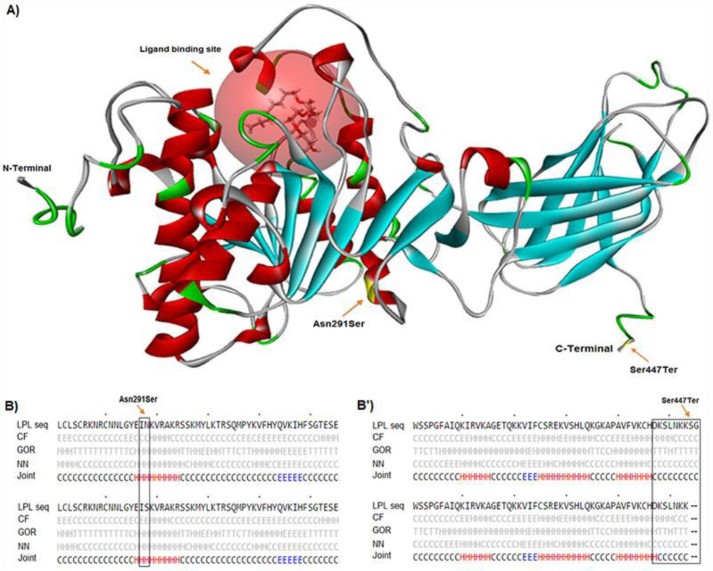
Tertiary and secondary structures of LPL. Asn291Ser and Ser447Ter are located at center and C-terminal of LPL, respectively (A). The secondary structures of LPL for 291Asn and 291Ser phenotypes are coil and extended β-strands, respectively (B). The secondary structure for Ser447Ter polymorphism changes in the GOR method (B’)

**Figure 5:**
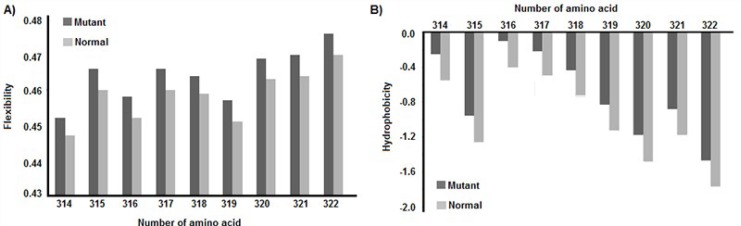
Hydrophobicity and average flexibility charts. After Asn291Ser substitution, the average flexibility (A) and hydrophobicity (B) of protein change in residues 314 to 322

**Figure 6:**
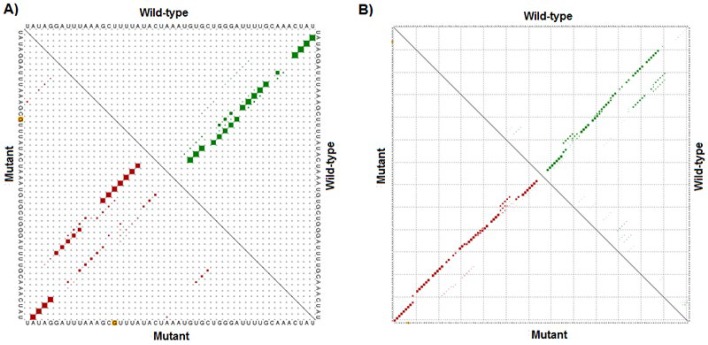
Results of RNAsnp. Enclosed area with marked differences in the mutant-type and wild-type LPL-hnRNA for rs320 (A) and rs285 (B). The possibility of wild-type and mutant sequences are illustrated in the top and bottom triangle of the graph, respectively. The polymorphic points are shown by yellow dots (A & B)

*Association of LPL-HindIII and LPL-PvuII with stroke risk*

The results of the meta-analysis about association of *LPL*-*Hind*III and stroke risk are summarized in Tables 2 and 3. When meta-analysis was performed for all five pooled studies, we observed a significant protective association between *LPL*-*Hind*III and stroke risk in G vs. T (OR= 0.64, 95%CI= 0.54-0.76, *P*<0.001), GG *vs*. TT (OR= 0.47, 95%CI= 0.29-0.75, *P*= 0.001), TG *vs*. TT (OR= 0.65, 95%CI= 0.53-0.80, *P*<0.001), TG+GG *vs*. TT (OR= 0.62, 95%CI= 0.51-0.75, *P*<0.001), and GG *vs*. TT+TG (OR= 0.51, 95%CI= 0.32-0.82, *P*=0.005) (Figure 2). These results resembled the results of the meta-analysis in the Chinese population which comprised four of the studies. In addition, the association between *LPL*-*Hind*III and stroke risk was further stratified by stroke subtypes, HWE status, country, and sample size. In the stratified analysis by stroke subtype, significantly decreased risks were observed for both hemorrhagic (G vs. T: OR= 0.58, 95%CI= 0.46-0.73, *P*<0.001; GG *vs*. TT: OR= 0.51, 95%CI= 0.27-0.94, *P*=0.031; TG *vs*. TT: OR= 0.57, 95%CI= 0.43-0.74, *P*<0.001; TG+GG *vs*. TT: OR=0.56, 95%CI= 0.43-0.72, *P*<0.001; GG *vs*. TT+TG; OR= 0.55, 95%CI= 0.30-1.03, *P*=0.061) and ischemic (G *vs*. T: OR= 0.72, 95%CI= 0.56-0.92, *P*= 0.010; GG *vs*. TT: OR= 0.42, 95%CI= 0.21-0.87, *P*= 0.019; TG+GG *vs*. TT: OR= 0.72, 95%CI= 0.54-0.98, *P*= 0.035; GG *vs*. TT+TG: OR= 0.46, 95%CI= 0.23-0.94, *P*= 0.033) strokes. The stratified analysis for HWE status showed that there is significant protective association between *Hind*III and risk of stroke in both studies with and without HWE. Moreover, significant decreased risk was observed among studies after stratifying by sample size (Table 2). 

Also, results of meta-analysis for association of *LPL*-*Pvu*II with stroke risk revealed that there is significant decreased risk in the four following models: P^-^ *vs*. P^+^ (OR= 0.72, 95%CI= 0.58-0.91, *P*= 0.005), P^-^P^-^ *vs*. P^+^P^+^ (OR= 0.50 95%CI= 0.31-0.82, *P*= 0.006), P^+^P^-^+P^-^P^-^ *vs*. P^+^P^+^ (OR= 0.72, 95% CI= 0.53-0.96, *P*= 0.027), P^-^P^-^ *vs*. P^+^P^+^+P^+^P^-^ (OR= 0.581, 95%CI= 0.369-0.916, *P*= 0.019). Even, after the adjusting of the *P*-values for multiple testing by the Benjamini-Hochberg false discovery rate method, both polymorphisms were still significantly associated with the stroke risk (Table 3).

*Heterogeneity, publication bias, and sensitivity analyses*

In the total meta-analysis, we found no true heterogeneity in any of the five genetic models for *Hind*III polymorphism. Also after a variety of stratifications, we did not observe any true heterogeneity (*P*_heterogeneity_> 0.1, Table 4). In addition, there was no significant heterogeneity for *Pvu*II polymorphism in any genetic models. 

Funnel plot and Egger’s test were used to measure the publication bias for the association of *Hind*III polymorphism and risk of stroke in the meta-analysis. Symmetrical shapes of funnel plot indicated lack of possible publication bias in the meta-analysis (Figure 3). Lack of publication bias was established by Egger’s test (Table 4). Exceptionally, publication bias was detected in the G *vs*. T model within the hemorrhagic subgroup (*P*_Egger_= 0.045), TG+GG *vs*. TT model in studies with HWE (*P*_Egger_= 0.037), and TG+GG *vs*. TT model in studies with sample size less than 500 participants (*P*_Egger_= 0.037). A sensitivity test was done by removing a study at a time. The results indicated that the estimations before and after the elimination of every study were alike, which suggests the outcome of the meta-analysis was stable (data not shown).

*In silico analysis*

We analyzed the effects of rs268, rs285, rs320, and rs328 polymorphisms on some molecular aspects of *LPL* such as RNA splicing, RNA stability, and function or structure of the protein by bioinformatics tools. Our data revealed that Asn291Ser substitution located near the ligand binding site of the protein (Figure 4) changes the secondary structure of LPL in the Choue-Fasman model. As illustrated in Figure 4, the secondary structures of LPL for 291Asn and 291Ser phenotypes are the coil and extended β-strand, respectively. Whereas, Ser447Ter polymorphism is located at the C-terminal of LPL and it changes the secondary structure of the protein in the GOR method (Figure 4). Also, Asn291Ser but not Ser447Ter substitution changes the hydrophobicity and average flexibility of the LPL protein. After Asn291Ser substitution, the hydrophobicity and average flexibility of protein change in residues 314 to 322 (Figure 5). Also, the SNAP web server predicted Asn291Ser as a damaging substitution (prediction: non-neutral; score: 19; expected accuracy: 59%). 

*In silico* analysis of *Pvu*II (rs285) and *Hind*III (rs320), polymorphisms showed that these mutations do not change the splicing pattern of *LPL* hnRNA. The NetGene2 data showed that there are no changes in donor and acceptor splice sites for either direct strand (+ strand) or complement strand (- strand) after rs285 and rs320 polymorphisms (**Figure S1**). Finally, we analyzed the impact of the two aforementioned SNPs on the RNA stability. The data from the RNAsnp software revealed that rs320 substitution has a significant effect on the structure of precursor RNA (distance: 0.2264, *P*-value: 0.0534; *P*<0.2 is a significant structural change). However, rs285 has no significant effect on the structure and stability of hnRNA (distance: 0.0657, *P*-value: 0.3495) (Figure 6).

## Discussion

Currently, stroke is a major clinical problem worldwide with high disability and mortality rates (27). Despite therapeutic improvement, prevention is an effective and economical strategy against stroke complication. Therefore, recognition of risk factors is very important. Several genetic factors are involved in the susceptibility to stroke (28, 29). Polymorphism of genes involved in lipid metabolism could be considered as a possible risk factor for stroke (30, 31). In this study, the association of rs285 and rs320 polymorphisms in the lipoprotein lipase gene with stroke risk was evaluated by meta-analysis. Our study revealed that *LPL*-*Hind*III is a protective factor against stroke risk in all of the five genetic models (*P*<0.05). Also, after stratification, we observed the same results for hemorrhagic and ischemic subtypes. The control groups in the studies of Xing *et al*. and Zhang *et al*. deviated from the Hardy-Weinberg principle (10, 24). But, exclusion of these two studies did not change the results of the meta-analysis. When the association analysis in studies with sample sizes less and more than 500 participants was performed, we observed the same significant protective association between *LPL*-*Hind*III and stroke risk. Regarding *LPL*-*Pvu*II polymorphism, we also observed a protective effect against the stroke risk. All of the included studies in the meta-analysis were from Asian populations of which four belonged to the Chinese population.

LPL is an important enzyme in lipoprotein metabolism, which hydrolyzes triglycerides in the core of high-fat lipoproteins such as chylomicron and VLDL. This enzyme is a 55-kDa transported to vascular lumen by glycosylphosphatidylinositol-anchored high-density lipoprotein-binding protein1 (GPIHBP1) and attached to the surface of vascular endothelium lumen by membrane heparin sulfate binding (32, 33). LPL activity is important for regulation of plasma triglyceride levels and supplies fatty acids for consumption in the heart and liver or storage in adipose tissue. Any deficiency in LPL activity leads to lipid metabolism defects such as hyperlipidemia and atherosclerosis, which are the main risk factors for myocardial infarction and stroke (1). Therefore, any variations in the protein and RNA sequence of LPL may change the enzyme activity and subsequent stroke susceptibility. SNPs could alter the gene expression, RNA structure, and protein function depending on their locations (34-36). Our previous studies revealed that *in silico* analysis is a useful approach to detect the effects of SNPs on RNA and protein structure (37-40).

In this study also the effect of common poly-morphisms (rs268, rs285, rs320, and rs328) on the molecular aspects of *LPL* has been investigated by *in silico* analysis. Our data revealed that rs285 and rs320 as intronic SNPs have no effect on splicing pattern of *LPL*, but rs320 has a significant impact on precursor RNA and it may be a source of the protective effect of rs320 in stroke susceptibility. Moreover, bioinformatics analysis showed rs268 and rs328 have a significant influence on the structure of the protein and this result could interpret the meta-analysis results of a previous study (11). 

The main limitation of this study is the restriction of included studies to Asian populations especially Chinese. Also, lack of access to original data such as familial history for stroke could affect the calculation of association between aforementioned SNPs and stroke risk.

## Conclusion

This meta-analysis suggests that the *LPL*-*Hind*III and *LPL*-*Pvu*II polymorphisms may alter the risk of stroke. But studies with larger sample sizes and different ethnicity are required to support these results. Also, studies with evaluation of gene-gene and gene-environment interactions are essential to achieving more accurate results.
